# The Prevalence of Age-Related Eye Diseases and Cataract Surgery among Older Adults in the City of Lodz, Poland

**DOI:** 10.1155/2015/605814

**Published:** 2015-02-19

**Authors:** Michal Szymon Nowak, Janusz Smigielski

**Affiliations:** ^1^Department of Ophthalmology and Visual Rehabilitation, Medical University of Lodz, 113 Zeromskiego Street, 90-549 Lodz, Poland; ^2^Department of Geriatrics, Medical University of Lodz, 1 Hallera Square, 90-647 Lodz, Poland

## Abstract

*Purpose.* To determine the prevalence of age-related eye diseases and cataract surgery among older adults in the city of Lodz, in central Poland. *Material and Methods.* The study design was cross-sectional and observational study. A total of 1107 women and men of predominantly Caucasian origin were successfully enumerated and recruited for the study. All selected subjects were interviewed and underwent detailed ophthalmic examinations. *Results.* Overall 8.04% (95% CI 6.44–9.64) subjects had cataract surgery in either eye. After excluding subjects with bilateral cataract surgery, the prevalence of cataract was 12.10% (95% CI 10.18–14.03). AMD was found in 4.33% (95% CI 3.14–5.54 ) of all subjects. Of them 3.25% (95% CI 2.21–4.30 ) had early AMD and 1.08% (95% CI 0.47–1.69) had late AMD. Various types of glaucoma were diagnosed in 5.51% (95% CI 4.17–6.85) of subjects and 2.62% (95% CI 1.68–3.56) had OHT. The prevalence rates of DR and myopic macular degeneration were 1.72% (95% CI 0.95–2.48) and 0.45% (95% CI 0.06–0.85), respectively. All multiple logistic regression models were only significantly associated with older age. The highest rate of visual impairment was observed among subjects with retinal diseases. *Conclusions.* The study revealed high prevalence of age-related eye diseases in this older population.

## 1. Introduction

The number of people with age-related eye diseases is assumed to be on the rise with increasing life expectancy [1]. Cataract is still the major cause of visual impairment and blindness globally, but other age-related eye diseases, that is, age-related macular degeneration (AMD), glaucoma, diabetic retinopathy (DR), and degenerative myopia, are becoming the most important causes in developed countries [2–7]. The most recent data published by World Health Organization (WHO) showed that the total number of persons with visual impairment worldwide in 2010 was estimated to be 285 million, including 39 million blind people, of whom around 80 per cent are above age of 50, with most of the causes being preventable [2]. In the European region alone there are 28 million visually impaired people, but there is a lack of data from Eastern Europe. The prevalence estimates of eye diseases from this part of the world are based on data collected mostly in the past century. One of the rare studies from Eastern Europe was performed in Bulgaria in 1995 [8]. In recent time not one comprehensive population-based ophthalmic survey conducted on elderly adults in postsoviet countries has been published and we decided to fill this gap.

Poland is the biggest postsoviet country in Eastern Europe with a population of 38.5 million people, of whom 15.9 million (41.3%) are aged 45 and older, according to 2011 national census [9]. The city of Lodz is situated in central Poland. Lodz consists of seven hundred forty thousand inhabitants and is the second biggest city in Poland, after Warsaw [9]. Because current healthcare costs have been rising, due to the ageing of the population, our study was also strongly motivated by the need to collect accurate prevalence data for the planning of specific prevention strategies for ophthalmic care in Poland.

The aim of our study was the assessment of the prevalence of age-related eye diseases such as cataract, AMD, glaucoma and ocular hypertension (OHT), DR, degenerative myopia, and cataract surgery in a sample population of Polish older adults.

## 2. Material and Methods

### 2.1. Subjects and Eye Examinations

The study design was a cross-sectional and observational epidemiological survey. The methodology of the subject sampling has been published in our previous studies. In brief, “sample size for the study was calculated with 99% confidence, within an error bound of 5%. The sample size requirement was 661, as calculated by

(1) $$\begin{array}{c} n=\frac{Z^{2}}{4d^{2}}, \end{array}$$

where *Z* = 2.57 for 99% confidence interval and *d* = 0.05 for 5% error bound. After allowing for an arbitrary 50% increase in sample size to accommodate possible inefficiencies associated with the sample design, the sample size requirement increased to 991 subjects [10, 11].” Since around the age of 40 physiological changes in the vision organ may be expected in our previous reports we considered young adult as person aged 18–34 years [10, 11]. For the current study we decided to define an older adult as person aged ≥ 35 years. We used simple systematic sampling to select our study population. In total 14110 outpatients were examined in the Department of Ophthalmology and Visual Rehabilitation of the Medical University of Lodz in the year 2012 and we included into the study every tenth subject aged 35 years and older. Based on age, the study participants were divided into two groups: group I aged 35–59 years, and group II aged 60 years and older. Information regarding the vision status, especially presence of one of the following diseases, cataract, glaucoma, age-related macular degeneration (AMD) diabetic retinopathy and/or diabetes, history of any cataract surgery, difficulties with distance, or near vision (presence of myopia), was assessed in all selected subjects on the same day as their scheduled visit in our outpatient department. Initial clinical examination included distance visual acuity (VA) testing, intraocular pressure (IOP) measurements using Goldman applanation tonometry, a cover test, and binocular and color vision assessments. VA was measured at a distance of 4 m, using a retroilluminated Snellen chart. Each eye was tested separately, with spectacles if worn. In all subjects who self-reported particular eye diseases (as mentioned above) and/or diabetes as well as in persons who had reduced VA (less than 0.5 decimal) mydriasis after administration of one drop of 1% cyclopentolate and slit lamp evaluation of the anterior and posterior segments using a 80 D Volk lens (supplied by Volk Optical, Mentor, OH, USA) were performed. As part of the ophthalmic examination cycloplegic refraction data were obtained only in eyes with distance visual acuity less than 0.5 decimal (<20/40) using the Topcon KR 8900 autorefractometer (supplied by Topcon Corporation, Tokyo, Japan). Based on this refraction, subjective refraction tests were performed to achieve best corrected visual acuity (BCVA). In the study subjects with the established diagnosis of age-related eye disorders further examinations were discontinued. However in all glaucoma suspects Medmont computerized perimetry (supplied by Medmont, Camberwell, Victoria, Australia) which was equivalent to 24.2 Humphrey visual field and disc analysis with Heidelberg Retina Tomograph II (HRT II, supplied by Heidelberg Engineering GmbH, Heidelberg, Germany) were scheduled and performed. Fluorescein angiography (FA) with Topcon TRC-50 EX fundus camera using Imagenet 2000 software (supplied by Topcon, Tokyo, Japan) and optical coherence tomography (OCT) with Topcon 3D OCT-1000 (supplied by Topcon, Tokyo, Japan) were scheduled and performed in the study participants if retinal diseases were suspected. All patient records were anonymized and deidentified prior to any data analysis, which included age socioeconomic status as well as brief details of the eye conditions. Ethical approval for this study was obtained from the institutional review board of the Medical University of Lodz and the work was conducted in accordance with the provisions of the Declaration of Helsinki for research involving human subjects.

### 2.2. Definitions

Visual impairment was defined as BCVA less than 0.5 decimal (<20/40) in either eye. Low vision was defined as BCVA less than 0.1 decimal (<20/40) but better than 0.1 decimal (>20/200) in better seeing eye and blindness was defined as BCVA equal to or less than 0.1 decimal (≤20/200) in both eyes (United States criteria) [5].

Age-related macular degeneration (AMD) was defined according to the international classification and grading system for age-related maculopathy and age-related macular degeneration developed by the International ARM Epidemiological Study Group [12]. In brief, early AMD was defined as a degenerative disorder characterized by the presence of the following abnormalities in the macular area: drusen, hyperpigmentation, and/or hypopigmentation of the retinal pigment epithelium (RPE). Late AMD was defined as the presence of geographic atrophy of the RPE (in the absence of neovascular AMD) or neovascular AMD defined as RPE and associated neurosensory detachment, retinal (periretinal) hemorrhages, and/or retinal (periretinal) fibrous scarring in the absence of other retinal (vascular) disorders [12].

Glaucoma was diagnosed based on characteristic morphological changes of the optic nerve head and retinal nerve fibre layer (RNFL) not related to other ocular diseases or congenital anomalies, associated with glaucomatous visual filed loss [13, 14]. When results of optic nerve head examinations and visual field were unavailable because of media opacity, glaucoma was diagnosed based on previous evidence of glaucoma treatment. The diagnosis of ocular hypertension (OHT) was based on elevated intraocular pressure with the remainder of the examination being normal [15].

Diabetic retinopathy (DR) was defined according to Early Treatment Diabetic Retinopathy Study Research Group [16]. In brief, DR was categorized into various stages of nonproliferative DR (NPDR) and proliferative DR upon presence of retinal hemorrhages and/or microaneurysms, venous beading, soft and hard exudates, new vessels, fibrous proliferations, and macular edema [16]. The diagnosis of diabetes mellitus was based on the use of diabetic medication.

The presence of myopic macular degeneration was assumed based on characteristic fundus appearance and FA and OCT examinations in patients with myopia.

The presence of cataract, aphakia or pseudophakia was determined upon the slit lamp examination. Cataract surgery was defined as the lens extraction in one or both eyes.

### 2.3. Data Management and Statistical Analysis

All statistical analyses were performed by using a commercially available software STATISTICA v. 10.1 PL (StatSoft Polska, Krakow, Poland). Prevalence rates of cataract, age-related macular degeneration (AMD), glaucoma, ocular hypertension (OHT), diabetic retinopathy (DR), degenerative myopia, and cataract surgery were estimated as percentage of all the participants. Multiple logistic regression statistics were used to investigate the association of cataract, AMD, glaucoma, and OHT as well as DR and degenerative myopia with age, gender, and socioeconomic status of participants. Each of them was independent model and odds ratios (ORs) were calculated. The prevalence of visual impairment, low vision, and blindness among subjects with age-related eye diseases was presented. Chi square tests were used for demographic analysis. All confidence intervals (CIs) in the study were 95% CI; differences were significant at *P* < 0.05.

## 3. Results

### 3.1. Subjects

A total of 1107 subjects were enumerated and successfully recruited for the study. All subjects were of European Caucasian origin. According to 2011 national census, they were a fair representation of the population of the city of Lodz in terms of sex distribution (statistical analysis, chi square test: *χ* ^2^ = 3.64, *P* > 0.05) and socioeconomic status [9]. The mean age of subjects included into the study was 60.4 ± 7.1 years (range, 35 to 97 years). Among all 1107 participants, 642 were women (58.0%) and 465 were men (42.0%). In addition, 520 (47.0%) subjects were aged 35–59 years, and 587 (53.0%) subjects were 60 years of age and older. In the researched population only 31 subjects (2.8%) declared to have no income; other socioeconomic factors, that is, marital status, level of education, or place of living, were not investigated. The sociodemographic characteristic of subjects and statistical analyses are presented in Table 1.

### 3.2. Prevalence of Age-Related Eye Diseases and Cataract Surgery

We obtained complete data in all of 1107 subjects. The most common age-related eye disease found in our study population was cataract. Data concerning the prevalence of age-related eye diseases are presented in Table 2. Overall 8.04% (95% CI 6.44–9.64) subjects had cataract surgery in at least one eye, and 6.05% (95% CI 4.65–7.46) had cataract surgery in both eyes. After excluding subjects with bilateral cataract surgery, the prevalence of cataract in at least one eye was 12.10% (95% CI 10.18–14.03) of all subjects. The prevalence of cataract increased with age from 2.69% (95% CI 1.30–4.08) in age group of 35–59 years to 20.44% (95% CI 17.18–23.71) in subjects aged ≥ 60 years. The prevalence of cataract was also higher in women. However, in a multiple logistic regression analysis with age, gender, and socioeconomic status, cataract was only associated with older age (OR 1.11, 95% CI 1.09–1.14).

AMD was found in 4.33% (95% CI 3.14–5.54) of all subjects. Of them 3.25% (95% CI 2.21–4.30) had early AMD and 1.08% (95% CI 0.47–1.69) had late AMD. The prevalence of AMD increased with age from 0.96% (95% CI 0.12–1.80) in age group of 35–59 years to 7.32% (95% CI 5.22–9.43) in group aged ≥ 60 years. A comparison between genders showed that the prevalence of AMD was similar in women and men. In multiple logistic regression analysis the prevalence of AMD was significantly associated with age (OR 1.10, 95% CI 1.07–1.13). Gender and socioeconomic status were not risk factors for AMD.

In total, glaucoma and ocular hypertension were diagnosed in 8.13% (95% CI 6.52–9.74) of subjects. Of them 5.51% (95% CI 4.17–6.85) had various types of glaucoma and 2.62% (95% CI 1.68–3.56) had ocular hypertension (OHT). The prevalence of glaucoma increased with age from 3.27% (95% CI 1.74–4.80) in age group of 35–59 years to 7.50% (95% CI 5.37–9.63) in subjects aged ≥ 60 years. The prevalence of OHT was also higher in subjects aged ≥ 60 years. In addition, the prevalence of both glaucoma and OHT was higher in women. However, multiple logistic regression analysis showed that the prevalence of glaucoma and OHT was only associated with age (OR 1.04, 95% CI 1.02–1.06). No association was found with gender and socioeconomic status of subjects.

DR was found in 1.72% (95% CI 0.95–2.48) of all subjects. The prevalence of DR was higher with increasing age and in men. The myopic macular degeneration was found in 0.45% (95% CI 0.06–0.85) of the study population. The prevalence of myopic macular degeneration was also higher in older age group. Since the number of people with DR and myopic macular degeneration was low, we combined these two groups together for analysis. In multiple logistic regression modelling with age, gender, and socioeconomic status, diabetic retinopathy and myopic macular degeneration were only associated with older age (OR 1.04, 95% CI 1.00–1.07).

### 3.3. Visual Acuity Distribution among Subjects with Age-Related Eye Diseases

Visual acuity findings are presented in Table 3. Totally visual impairment in at least one eye was found in 39 (13.18%) subjects with age-related eye diseases. Of them 15 subjects had visual impairment in both eyes, which accounted for 5.06% of whole population. This included 12 (4.05%) subjects with low vision and 3 (1.01%) subjects with bilateral blindness. Among those with myopic macular degeneration 40% had visual impairment and 20% were blind. Other causes of blindness were AMD and glaucoma. Following myopic macular degeneration, the prevalence of visual impairment in at least one eye was highest in AMD cases and the prevalence of bilateral visual impairment was highest among DR cases. The lowest number of cases with visual impairment was among subjects presenting with cataract.

## 4. Discussion

Although many studies concerning the prevalence of ocular diseases in elderly populations have been recently conducted worldwide [1, 17–20], this is the first such study from Eastern Europe. We assessed information on the prevalence of clinically relevant cataract, AMD, glaucoma and OHT, DR, and myopic macular degeneration as well as on the prevalence of cataract surgery in Polish citizens aged 35 years and older. When combined together the overall prevalence of any cataract or cataract surgery was 20.14%. The prevalence of cataract alone was 12.10% in the whole population, but in age group ≥ 60 years it increased to 20.44% and was significantly associated with older age. Direct comparison of our results to the results from other studies conducted on elderly populations is limited due to the fact that other studies comprised different age groups. Other possible limitations include different methodology of the ophthalmic examination and population sampling. However the overall prevalence of cataract in our study was lower than in studies from Australia, South-East Asia, and North America [18–22], but it was higher than what was found in population aged ≥ 30 years in Finland [1]. We did not find any association between cataract and gender or socioeconomic status of subjects, but the results of previous studies revealed that other factors associated with cataract included myopia, diabetes, asthma, cardiovascular diseases, cigarette smoking, and female gender [1, 21, 23]. The prevalence rate of cataract surgery in either eye in Poland was similar to that found in SiMES-1 study in Singapore [20] and was higher than that found in Finland, United States, and Australia [1, 18, 22]. In addition it was significantly higher than the prevalence rate of cataract surgeries found in India and in rural or urban China [19, 21, 24]. The high rate of cataract surgery in Poland is a proof of rapid economic development in postsoviet era, but in some Eastern European countries the number of cataract surgeries per million population per year might be significantly lower than in Western Europe [25].

AMD is the leading cause of visual impairment and blindness in western countries [26]. Our results showed that the total prevalence of AMD among older adults in Poland was 4.33%. This included 3.25% of subjects with early AMD and 1.08% subjects with late AMD. The prevalence of AMD in our study population was rather low, and this was mainly attributable to the infrequent existence of AMD (0.96%) in the age group 35–59 years. In those aged ≥ 60 years the prevalence of AMD increased to more than 7.32%. Findings from our study were in agreement with research performed by Laitinen et al., who found AMD in 3.8% of subjects in a representative sample of the adult Finnish population aged ≥ 30 years [1]. On the basis of available reports the prevalence of AMD varied with age and ethnicity from 1.5% among older adults in the Bhaktapur district in Nepal to 38.4% among adults aged 65–87 years in the municipality of Tromsø, Norway [27, 28], and it was higher in Caucasians than in non-Caucasians and African Americans [18–20, 29]. The prevalence rate of late AMD in Poland was not markedly different than that found in other studies performed on predominantly Caucasian populations [29–31]. In the present study multiple logistic regression analyses showed that age-related macular degeneration was only associated with age. Our results were in agreement with the results from previous studies which have reported no gender specific differences of the prevalence of AMD [32]. However some studies have reported the prevalence of neovascular AMD to be higher in women [33]. Other studies also confirmed that the prevalence of AMD is strongly associated with cigarette smoking and with some cardiovascular risk factors [34].

The total prevalence of glaucoma and ocular hypertension in our study population was rather high (8.13%). Various types of glaucoma were found in 5.51% and ocular hypertension (OHT) was found in 2.61% of all subjects. However there is no single standard for defining glaucoma in population based research and our results were closer to the rates of glaucoma found among older adults in Finland and Singapore and among adult Latinos in the USA [1, 20, 35]. The prevalence of glaucoma in Poland was higher than in several previous studies conducted on subjects of predominantly European Caucasian origin outside Europe [36, 37] but it was lower than what was found in Caucasian populations in Germany and in the European North of Russia [17]. In addition the prevalence of OHT in Poland was also lower than what was found in other studies [19, 35]. The multiple logistic regression analysis showed that glaucoma and OHT were only associated with older age. Despite the fact that the link between sex and glaucoma has been investigated with contradictory results, our findings were similar to the studies which did not find gender as a significant risk predictor [20, 35].

Glaucoma is one of the leading causes of world blindness, especially in Asians, and the prevalence of glaucoma is going to rise substantially due to the ageing of populations [2, 5, 38]. Although the estimated future prevalence of open-angle glaucoma is highest in Africa and of angle-closure glaucoma in Asia [38], the results of our study revealed that we should also be aware about the growing number of glaucoma patients in Poland.

Diabetic retinopathy (DR) is one of the most important causes of low vision and blindness in the working age population globally [17, 18, 39] and myopic macular degeneration is the leading cause of severe visual impairment in Japan and China [5, 19]. The prevalence of DR and myopic macular degeneration in Poland was 1.72% and 0.45%, respectively. Our prevalence rate of DR was higher than what was found in subjects of predominantly Caucasian origin in the National Health Interview Survey (NHIS) in the United States and among adults in Finland, but those studies comprised younger age groups [1, 18]. It was also higher than rates found in China and Nepal [27, 39]. On the other hand it was lower than DR prevalence found in Australia and Singapore and in other studies from the United States [18, 20, 40, 41]. The results of previous WHO survey also revealed that the incidence of any DR in patients with type II diabetes in Poland was 31.8% [42]. The prevalence of DR in our study was higher in older age group and in men but in multiple logistic regression modeling with age, gender, and socioeconomic status of subjects, only increasing age was an independent risk factor associated with DR and myopic macular degeneration.

Overall the prevalence of low vision and blindness among subjects with age-related eye diseases was higher than what was found in this age group in Poland and in other populations in Europe [3, 6, 7]. In the present study the highest rate of visual impairment was among subjects with myopic macular degeneration. Although myopic macular degeneration has been rarely reported as a major cause of impaired vision in white persons, it was the predominant cause of severe visual loss among subjects younger than 75 years in the Rotterdam Study in the Netherlands [7]. Our findings were also in agreement with the results of previous studies which showed high prevalence of visual impairment among AMD and DR subjects of predominantly Caucasian origin in Western Europe [1, 3, 17, 28]. The number of cases with bilateral visual impairment in our study was the lowest in subjects presenting with cataract. This emphasises the role of cataract surgery in the prevention or treatment of possible blindness in Poland and worldwide.

## 5. Conclusions

This study provides for the first time a summary of the prevalence of clinically relevant age-related eye diseases among older adults in Poland. Although the most common condition was age-related cataract, the highest rate of visual impairment was observed among subjects with retinal diseases. The study also revealed high prevalence of age-related eye diseases in this older population.

## Figures and Tables

**(a):** 

Examined group	Number of subjects: *n* (%)	Min	Max	Mean	Med	Std. dev.	35–59 years	≥60 years	Have any source of income	Have no income
All	1107 (100%)	35.0	97.0	60.4	61.0	12.8	520 (100%)	587 (100%)	1076 (97.2%)	31 (2.8%)
Men	465 (42.0%)	35.0	97.0	59.8	60.0	14.1	230 (44.2%)	235 (40.0%)	452 (97.2%)	13 (2.8%)
Women	642 (58.0%)	35.0	93.0	60.7	61.0	11.7	290 (55.8%)	352 (60.0%)	624 (97.2%)	18 (2.8%)

							Chi square *P* = 0,158		Chi square *P* = 0,994	

**(b):** 

Examined group	Number of subjects: *n* (%)	Min	Max	Mean	Med	Std. dev.	Men	Women	Have any source of income	Have no income
All	1107 (100%)	35.0	97.0	60.4	61.0	12.8	465 (100%)	642 (100%)	1076 (97.2%)	31 (2.8%)
35–59 years	520 (47.0%)	35.0	59.0	49.3	50.0	7.1	230 (49.5%)	290 (45.2%)	500 (96.1%)	20 (3.9%)
≥60 years	587 (53.0%)	60.0	97.0	70.1	69.0	7.8	235 (50.5%)	352 (54.8%)	576 (98.1%)	11 (1.9%)

							Chi square *P* = 0,158		Chi square *P* = 0,047	

**Table 2:** The prevalence of age-related eye diseases in the study population.

	35–59 years	≥60 years	Men	Women	All
	*n* (%; 95% CI)	*n* (%; 95% CI)	*n* (%; 95% CI)	*n* (%; 95% CI)	*n* (%; 95% CI)
Cataract	14 (2.69%; 1.30–4.08)	120 (20.44%; 17.18–23.71)	53 (9.25%; 8.51–14.29)	81 (12.62%; 10.05–15.19)	134 (12.10%; 10.18–14.03)
Age-related macular degeneration (AMD)	5 (0.96%; 0.12–1.80)	43 (7.32%; 5.22–9.43)	20 (4.30%; 2.46–6.15)	28 (4.36%; 2.78–5.94)	48 (4.33%; 3.14–5.54)
Early AMD	4 (0.77%; 0.02–1.52)	32 (5.45%; 3.61–7.29)	14 (3.01%; 1.46–4.56)	22 (3.47%; 2.02–4.83)	36 (3.25%; 2.21–4.30)
Late AMD	1 (0.19%; 0.00–0.57)	11 (1.87%; 0.78–2.97)	5 (1.07%; 0.14–2.01)	7 (1.09%; 0.29–1.89)	12 (1.08%; 0.47–1.69)
Glaucoma	17 (3.27%; 1.74–4.80)	44 (7.50%; 5.37–9.63)	21 (4.52%; 2.63–6.40)	40 (6.23%; 4.36–8.10)	61 (5.51%; 4.17–6.85)
Ocular hypertension	11 (2.11%; 0.88–3.35)	18 (3.07%; 1.67–4.46)	10 (2.15%; 0.83–3.47)	19 (2.96%; 1.65–4.27)	29 (2.62%; 1.68–3.56)
Diabetic retinopathy	3 (0.58%; 0.00–1.23)	16 (2.73%; 1.41–4.04)	10 (2.15%; 0.83–3.47)	9 (1.40%; 0.49–2.31)	19 (1.72%; 0.95–2.48)
Degenerative myopia	1 (0.19%; 0.00–0.57)	4 (0.68%; 0.02–1.35)	2 (0.43%; 0.00–1.02)	3 (0.47%; 0.00–0.99)	5 (0.45%; 0.06–0.85)

All	520 (100%)	587 (100%)	465 (100%)	642 (100%)	1107 (100%)

**Table 3:** Visual acuity distribution among subjects with age-related eye diseases^*^.

	Number of subjects *n* (%)	No impairment *n* (%)	Visual impairment in at least one eye *n* (%)	Visual impairment in both eyes *n* (%)	Low vision *n* (%)	Blindness *n* (%)
Cataract	134 (100%)	122 (91.05%)	12 (8.95%)	3 (2.24%)	3 (2.24%)	0 (0.00%)
Age-related macular degeneration (AMD)	48 (100%)	34 (70.83%)	14 (29.17%)	5 (10.41%)	4 (8.33%)	1 (2.08%)
Glaucoma and ocular hypertension	90 (100%)	84 (93.33%)	6 (6.67%)	3 (3.33%)	2 (2.22%)	1 (1.11%)
Diabetic retinopathy	19 (100%)	14 (73.68%)	5 (26.32)	2 (10.53%)	2 (10.53%)	0 (0.00%)
Degenerative myopia	5 (100%)	3 (60.00%)	2 (40.00%)	2 (40%)	1 (20.00%)	1 (20.00%)

All	296 (100%)	257 (86.82)	39 (13.18)	15 (5.06%)	12 (4.05%)	3 (1.01%)

^*^Visual impairment was defined as distance visual acuity <20/40 in either eye. Low vision was defined as best corrected visual acuity (BCVA) <20/40 but >20/200 in better-seeing eye and blindness was defined as BCVA ≤20/200 in both eyes (United States criteria).
